# Supplementary material for: Imaging the electrical activity of organelles in living cells
Source: Commun Biol. 2021 Mar 23;4:389. doi: 10.1038/s42003-021-01916-6 (PMC7988155; doi:10.1038/s42003-021-01916-6)
Supplement: Supplementary file 2 — Supplementary Information [file 42003_2021_1916_MOESM2_ESM.pdf]

IMAGING THE ELECTRICAL ACTIVITY OF ORGANELLES IN LIVING CELLS

Ella Matamala, Cristian Castillo, Juan P. Vivar, Patricio A. Rojas, and Sebastian E. Brauchi

SUPPLEMENTARY MATERIAL

3 supplementary figures

Supplementary figures

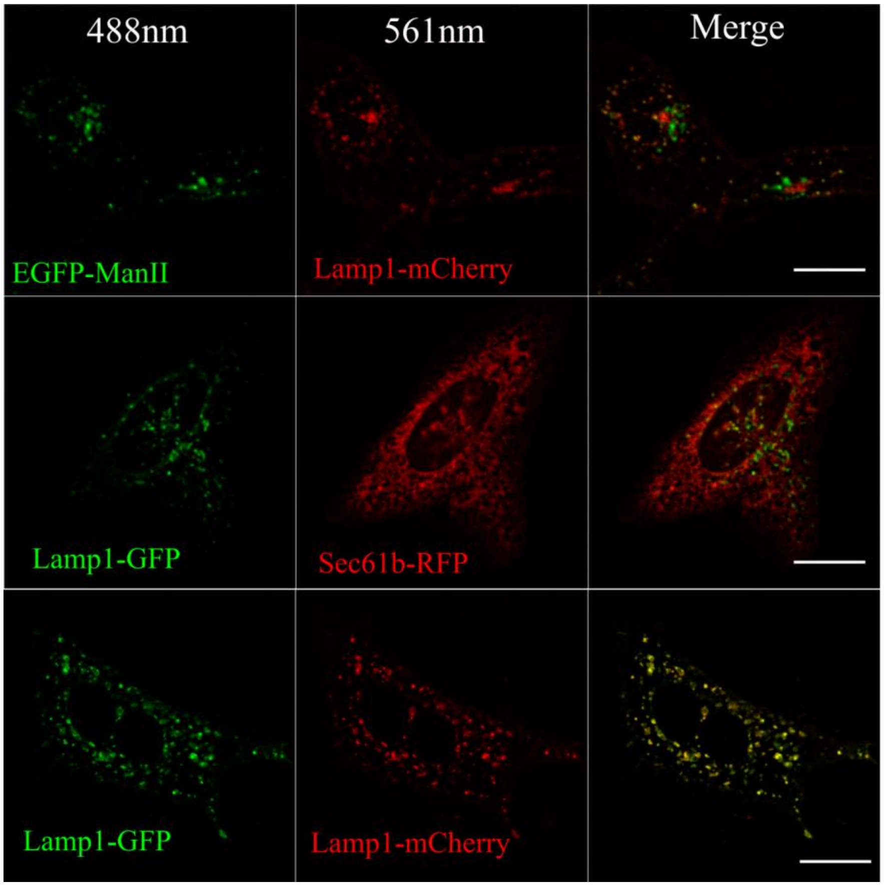

Supplementary figure 1.

Imaging of sub-cellular markers. Representative colocalization images of the lysosomal marker Lamp1 with the organellar markers EGFP-ManIIa (Golgi) and Sec61b-Cherry (ER). Extensive colocalization is observed in cells co-expressing two differently tagged Lamp1 markers (EGFP/mCherry). Scale bars correspond to 5 μm.

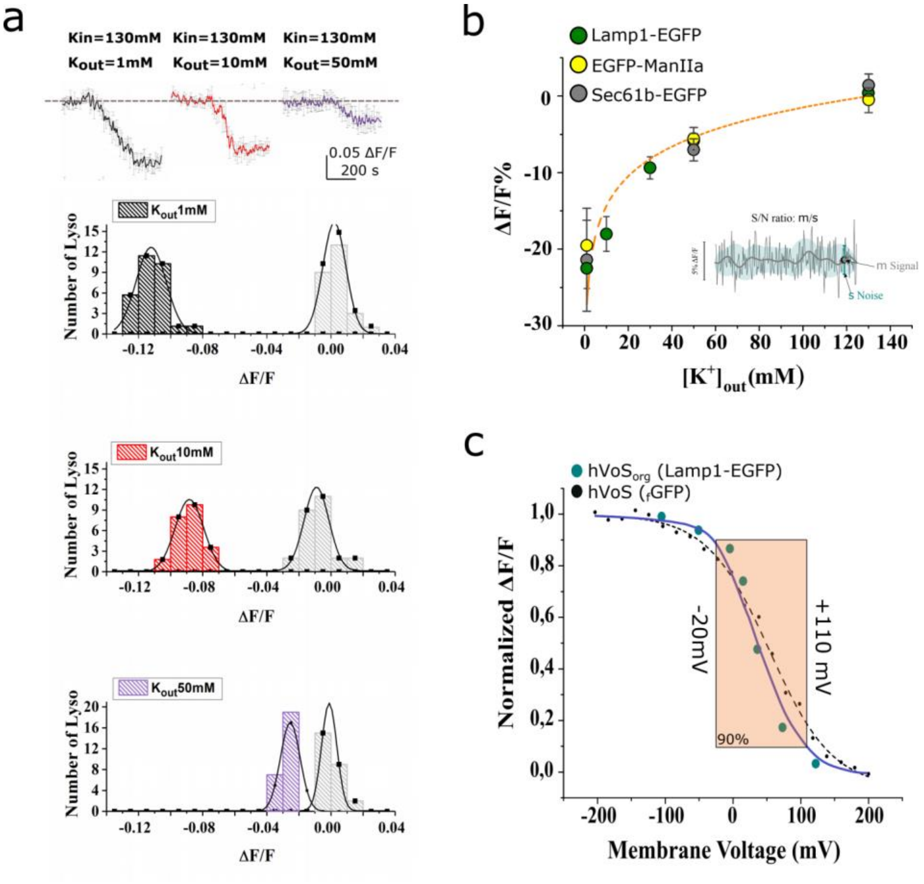

Supplementary figure 2.

Calibration procedure and the hVoS$_{org}$ response. (a) Representative traces and histograms used during the calibration procedure. Data represents one single experiment in which we observed the paired response of more than 20 lysosomes per condition. (b) Changes in fractional fluorescence are similar for all the markers used in this study. Inset: unfiltered trace depicting the signal and noise of our recordings from individual organelles. (c) Normalized fractional fluorescence versus membrane potential comparing previously published data for hVoS expressed at the plasma membrane [19] and the calibration curve obtained by in-cell potassium clamp.

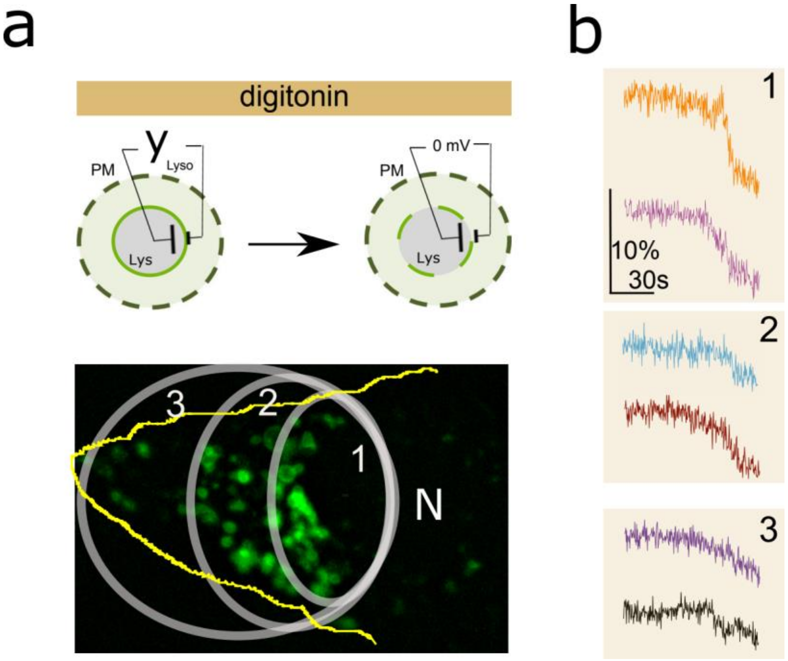

Supplementary figure 3.

Peripheral lysosomes are less depolarized. (a) Top: Cartoon on top depicts the experimental procedure. Digitonin permeabilization of the lysosomal membrane will dissipate all the membrane potential at each individual structure. Bottom: Image of a cell transiently expressing Lamp1-EGFP. The yellow line indicates the cell's plasma membrane and N denotes the position of the nucleus. Semicircular areas were defined and numbered. (b) Representative traces obtained from single lysosomes. Traces are grouped according to the areas defined in a.
