# Supplementary material for: Imaging the electrical activity of organelles in living cells
Source: Commun Biol. 2021 Mar 23;4:389. doi: 10.1038/s42003-021-01916-6 (PMC7988155; doi:10.1038/s42003-021-01916-6)
Supplement: Supplementary file 3 — Description of Additional Supplementary Files [file 42003_2021_1916_MOESM3_ESM.pdf]

Description of Additional Supplementary Files

File name: Supplementary Movie 1

Description: One cell exposed to digitonin. ΔF/F signal in pseudo color for a cell expressing hVoSorg and treated with digitonin. Digitonin was added in frame 10

File name: Supplementary Movie 2

Description: One cell exposed to rapamycin. ΔF/F signal in pseudo color for a cell expressing hVoSorg and exposed to rapamycin. Rapamycin was added in frame 10.

File name: Supplementary Data 1

Description: Supplementary Data 1. Annex1. Multi compartment model describing DPA distribution within membrane compartments implemented as a series of differential equations written in Python language. The original file and source data is available in the Github repository (https://github.com/brauchilab/DPA-quenching-model).

File name: Supplementary Data 2

Description: Source data used to fit the mathematical model.
