# Supplementary material for: Imaging the electrical activity of organelles in living cells
Source: Commun Biol. 2021 Mar 23;4:389. doi: 10.1038/s42003-021-01916-6 (PMC7988155; doi:10.1038/s42003-021-01916-6)

Annex 1- DPA quenching¶

A multi compartment model consisting of an extracellular reservoir (E), an extracellular (M1) and intracellular membrane leaflets (M2), and a cytosolic compartment (C) was implemented as a series of differential equations in Python language. Rate constant from extracellular and intracellular compartments to membrane were the same (a), and rates from membrane to both extracellular and intracellular compartments were the same (b) (Cooper et al., 1981). Rates between the two membrane leaflets (v1,v2) were obtained by extracting time constants for charge movement from whole-cell membrane capacitance measurements in presence of 4 µM DPA in mast cells (Oberhauser & Fernandez, 1995). Time constants in function of voltage follow a bell-shaped curve, then we fitted from negative voltages to the peak with the equation:

$v2 = B0e\text{^}(-slp2V*F/RT)$

while data from peak towards positive voltages was fitted to:

$v1 = A0exp(-slp1V*F/RT)$

From the fit we obtained A0, B0, slp1, slp2, which were used for the model. R is the gas constant, T is the absolute temperature and, F is the Faraday constant, V was assumed -30 mV. Fluorescence quenching was fitted to the model to obtain rate constants a and b, and parameters A0, B0, slp1, slp2 to calculate rate constants v1 and v2. With all this rates we run the model to calculate probabilities to find molecules in each compartment. By assuming DPA density at equilibrium 10-4 molecules/A2 (Oberhauser & Fernandez 1995), a cell with diameter of 40 µm and a membrane width of 10 nm, we were able to transform probabilities to concentration in membrane and cytosol.

Cooper et al. (1981) https://doi.org/10.1002/jps.2600700110
Oberhauser & Fernandez (1995) https://dx.doi.org/10.1016%2FS0006-3495(95)79918-0

In [2]:

```
import numpy as np
from scipy import integrate
import matplotlib.pyplot as plt
import time as tm
from scipy.optimize import curve_fit
from scipy.stats import mannwhitneyu
from scipy.signal import savgol_filter
import matplotlib.gridspec as gridspec
import math

# This file has corrected values of fitted
parameters
```

Delete points before DPA application

In [3]:

```
# remove baseline before fitting
trazos_pm   = -np.genfromtxt("pm_raw.csv",
delimiter=',')
t_trazos= np.linspace(0, np.shape(trazos_pm)[0],
np.shape(trazos_pm)[0])
#t_dele = [130,90,110,95]
t_dele = [115,100,100,100]
#dele = range(t_dele)
plt.figure(figsize=(10,10))

for i in range(4):
    plt.subplot(2,2,i+1)
    plt.plot(t_trazos,trazos_pm[:,i], "b", label=
"original")
    plt.axvline(t_dele[i], color ="k", ls="--")
    dele = range(t_dele[i])
    trazos_pm_d = np.delete(trazos_pm, dele,
axis=0 )
    t_trazos_d= np.linspace(0,
np.shape(trazos_pm_d)[0], np.shape(trazos_pm_d)[0])
    plt.plot(t_trazos_d,trazos_pm_d[:,i],
"k",label= "chopped")
```

```
    tit_str = "trace "+str(i)+"\n starts at time
"+str(t_dele[i])
    plt.title(tit_str)
    plt.grid()
    plt.legend()
plt.suptitle("delete points before fitting")
#plt.savefig("fig2.png")
```

Out[3]:

```
Text(0.5,0.98,'delete points before fitting')
```

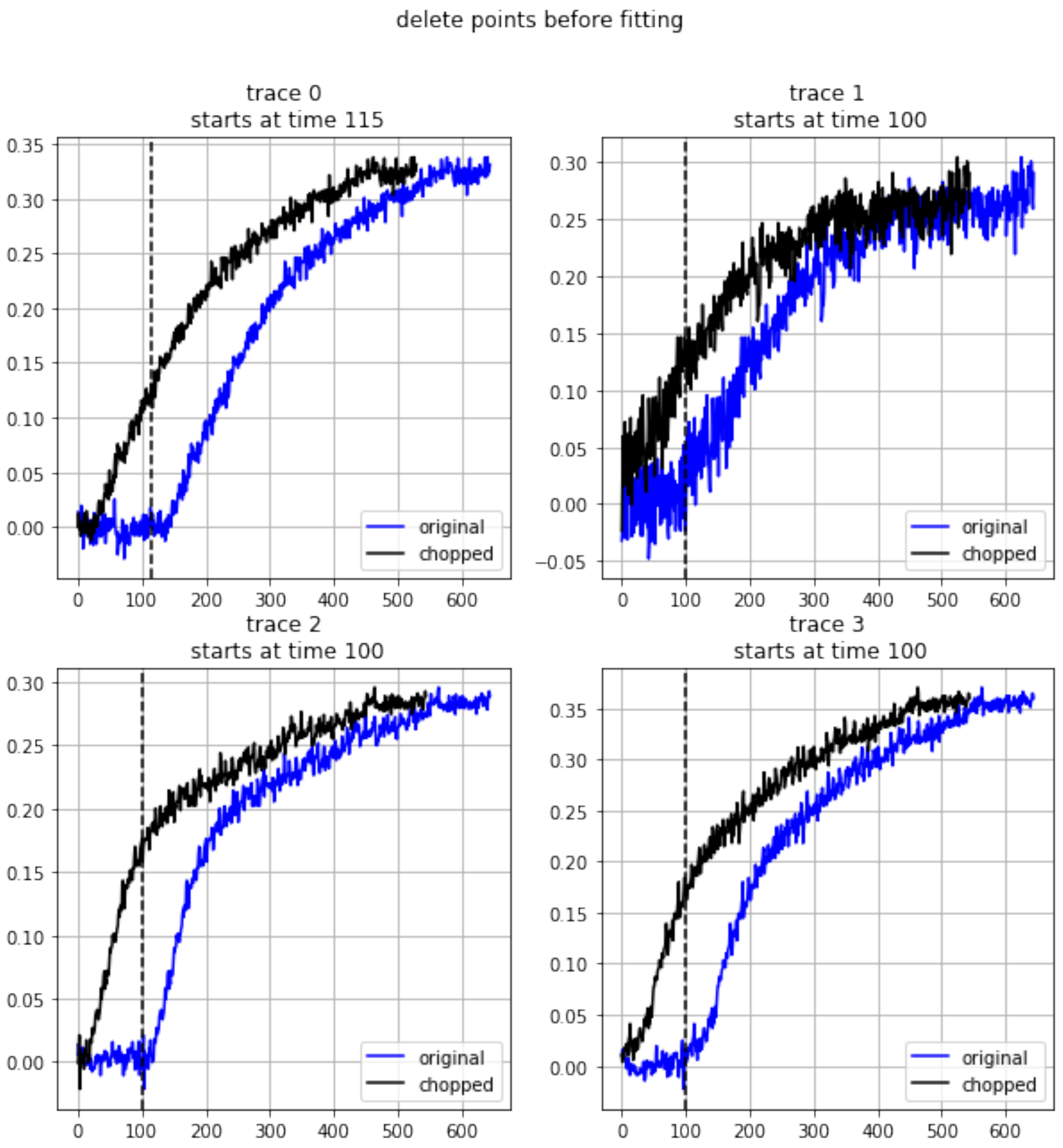

Kinetic model is

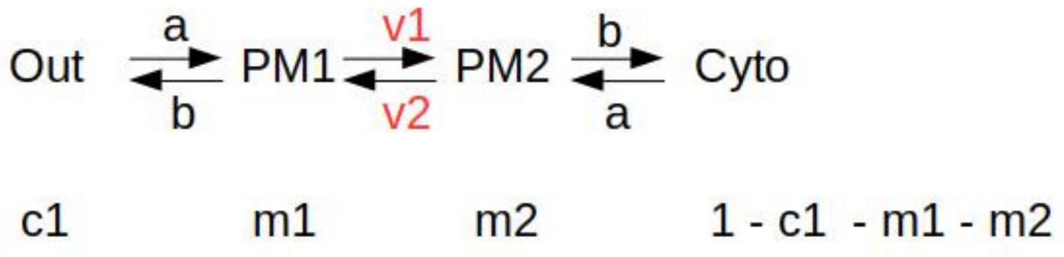

In [4]:

```
### fit data to four compartment model, one v-dep
transition (PM)
#
# Function to fit the model
def y(t, a,b):
    def f(z, t, a,b):  # Model , a and b are rate
constant to be fitted
        c1,m1,m2 = z   # Probability to be in each
compartment, can be transformed in concentration
        A0 = 10643.1
        slp_a = -.215
        V = -30  # membrane potential
        v2 = A0*np.exp(-slp_a*V/58) # .002 #
voltage dependent rate constant
        B0 = 8624.9
        slp_b = .278
        v1 = B0*np.exp((1-slp_b)*V/58) # .002 #
voltage dependent rate constant

        dydt = [-a*c1 + b*m1,              # C1
            a*c1 + v2*m2 - b*m1 - v1*m1, # M1
            v1*m1 + a*(1- c1 -m1 -m2) - v2*m2
-b*m2]  # M2
            #b*m2 -a*(1- c1 -m1 -m2)]      # Cyto
        return dydt
    y0=[1,0,0]  # initial condition for each
compartment
    y = integrate.odeint(f, y0, t, args=(a,b))  #
```

```
solve ODE
    return y[:,1] # the output is m2

trazos_pm   = -np.genfromtxt("pm_raw.csv",
delimiter=',')
t_trazos= np.linspace(0, np.shape(trazos_pm)[0],
np.shape(trazos_pm)[0]) # time array
t_dele = [130,90,110,95]    # point numbers to be
removed for each experiment
t_dele = [115,100,100,100]  # point numbers to be
removed for each experiment
#dele = range(t_dele)
a,b = [], []
#tr_0, tr_1, tr_2, tr_3 =[], [], [], []
#tr = [tr_0, tr_1, tr_2, tr_3]
plt.figure(figsize=(10,10))
print("    a            b")
#--fit traces to model and plot---
for i in range(4):
    plt.subplot(2,2,i+1)
    #plt.plot(t_trazos,trazos_pm[:,i], "b", label=
"original")
    dele = range(t_dele[i])  # array of indeces to
be removed
    #trazos_pm_d = np.delete(trazos_pm[:,i], dele,
axis=0 )
    # remove inital data points
    trazos_pm_d = np.delete(trazos_pm, dele,
axis=0 )
    # create time array
    t_trazos_d= np.linspace(0,
np.shape(trazos_pm_d)[0], np.shape(trazos_pm_d)[0])
    #print (np.shape(trazos_pm_d),
np.shape(t_trazos_d))
    # plot data
    plt.plot(t_trazos_d,trazos_pm_d[:,i],
"k",label= "data")
    # fit data to model with guesses (p0)
```

```
    popt, cov = curve_fit(y, t_trazos_d,
trazos_pm_d[:,i], p0 = [0.006,0.002] )
                         #bounds= ((0.002,0.001,0),
(.01,.009,1)))
    # plot fitting
    plt.plot(t_trazos_d, y(t_trazos_d, *popt), '-
r', label="ODE fit")
    print("%.6f" % popt[0] +"     " +"%.6f" %
popt[1])
    a.append(popt[0])
    b.append(popt[1])
    tit_str = "trace "+str(i) #+"\n starts at time
"+str(t_dele[i])
    plt.title(tit_str)
    txt_fit = "on: %.4f" % popt[0] + "\noff: %.4f"
% popt[1]
    plt.text(500, .1,txt_fit,
horizontalalignment="right") # , weight = 'bold' #
y_txt[i]
    plt.grid()
    plt.legend()
plt.suptitle("Quenching in plasma membrane")
print("------------------------------")
print("%.6f" %  np.mean(a) +"     "+ "%.6f   mean"
% np.mean(b))
sd_a = np.std(a)/np.sqrt(4)
sd_b = np.std(b)/np.sqrt(4)
print("%.6f" %  sd_a +"     " "%.6f   sd" % sd_b)
print("------------------------------")
print("------------------------------")
print(" a : %.4f" %  np.mean(a) + " +- %.4f" %
(np.std(a)/np.sqrt(4)))
print(" b : %.4f" %  np.mean(b) + " +- %.4f" %
(np.std(b)/np.sqrt(4)))
#plt.savefig("ODE_fit_pm.png")
```

```
    a            b
0.002343     0.001253
```

```
0.002774     0.003267
0.003574     0.004223
0.003419     0.002193
------------------------------
0.003028     0.002734   mean
0.000248     0.000558   sd
------------------------------
------------------------------
 a : 0.0030 +- 0.0002
 b : 0.0027 +- 0.0006
```

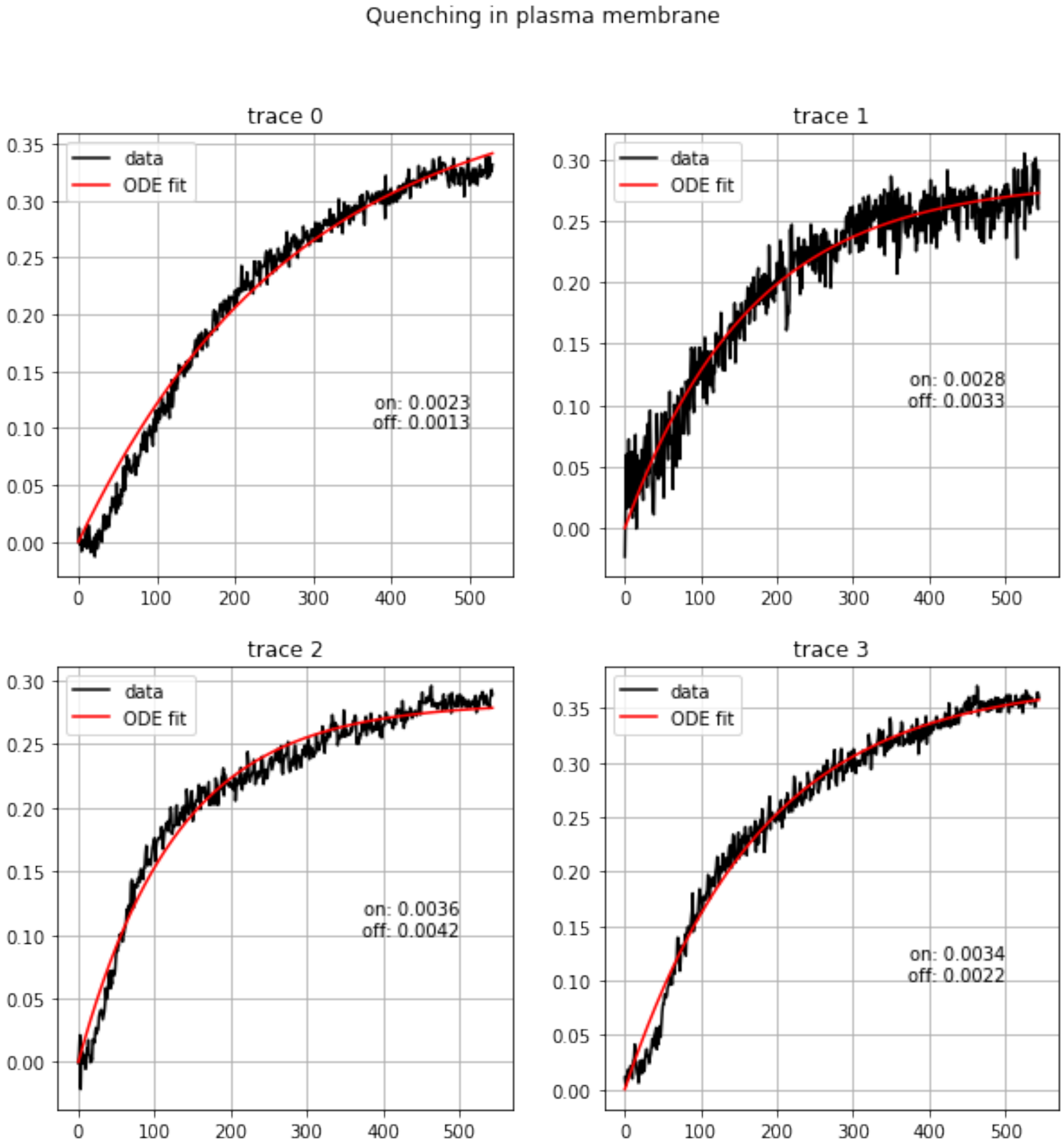

Fitting to different membrane potentials

In [5]:

```
### fit data to six compartment model  (v-dep)
# transition within plasma membrane is voltage
dependent
#---Model----
def y(t, a,b):
    def f(z, t, a,b,j):
        c1,m1,m2 = z
        A0 = 10643.1
        slp_a = -.215
        volt = np.arange(-100,100,20)
        v2 = A0*np.exp(-slp_a*volt[j]/58) # .002 #
        B0 = 8624.9
        slp_b = .278
        v1 = B0*np.exp((1-slp_b)*volt[j]/58) # .002
#

        dydt = [-a*c1 + b*m1,                # C1
            a*c1 + v2*m2 - b*m1 - v1*m1, # M1
            v1*m1 + a*(1- c1 -m1 -m2) - v2*m2
-b*m2]  # M2
            #b*m2 -a*(1- c1 -m1 -m2)]      # Cyto
        return dydt
    y0=[1,0,0]
    y = integrate.odeint(f, y0, t, args=(a,b,j))
    return y[:,2] #

trazos_pm   = -np.genfromtxt("pm_raw.csv",
delimiter=',')
#t_trazos= np.linspace(0, np.shape(trazos_pm)[0],
np.shape(trazos_pm)[0])
t_dele = [130,90,110,95]
t_dele = [115,100,100,100]
#dele = range(t_dele)
#a,b = [], []
#tr_0, tr_1, tr_2, tr_3 =[], [], [], []
```

```
#tr = [tr_0, tr_1, tr_2, tr_3]
plt.figure(figsize=(10,10))
#print(" \t\ta  \tb \tr^2")
#
#--fit traces to model and plot---
volt = np.arange(-100,100,20)
for i in range(4):
    plt.subplot(2,2,i+1)
    #plt.plot(t_trazos,trazos_pm[:,i], "b", label=
"original")
    dele = range(t_dele[i])
    #trazos_pm_d = np.delete(trazos_pm[:,i], dele,
axis=0 )
    trazos_pm_d = np.delete(trazos_pm, dele,
axis=0 )
    t_trazos_d= np.linspace(0,
np.shape(trazos_pm_d)[0], np.shape(trazos_pm_d)[0])
    #print (np.shape(trazos_pm_d),
np.shape(t_trazos_d))
    plt.plot(t_trazos_d,trazos_pm_d[:,i],
"k",label= "data")
    #--fit to a range of voltages------
    for j in range(len(volt)):
        popt, cov = curve_fit(y, t_trazos_d,
trazos_pm_d[:,i], p0 = [0.006,0.002] )
                                #bounds=
((0.002,0.001,0),(.01,.009,1)))
        plt.plot(t_trazos_d, y(t_trazos_d, *popt),
label=str(volt[j]))
        # calculate R squared
        residuals = trazos_pm_d[:,i] -
y(t_trazos_d, *popt)  # residuals
        ss_res = np.sum(residuals**2)
        ss_tot = np.sum((trazos_pm_d[:,i]
-np.mean(trazos_pm_d[:,i]))**2)
        r_squared =  1 - (ss_res / ss_tot)
        #print(str(volt[j]) + " mV" +"\t\t%.4f" %
popt[0] +"\t%.4f" % popt[1]
```

```
             #+ "\t%.4f" % r_squared)
    #a.append(popt[0])
    #b.append(popt[1])
    #print("----")
    tit_str = "trace "+str(i) #+"\n starts at time
"+str(t_dele[i])
    plt.title(tit_str)
    #txt_fit = "on: %.4f" % popt[0] + "\noff: %.4f"
% popt[1]
    #plt.text(500, .1,txt_fit,
horizontalalignment="right") # , weight = 'bold' #
y_txt[i]
    plt.grid()
    plt.legend()
plt.suptitle("Quenching in plasma membrane")
#plt.savefig("ODE_fit_pm_v_dep.png")
```

Out[5]:

```
Text(0.5,0.98,'Quenching in plasma membrane')
```

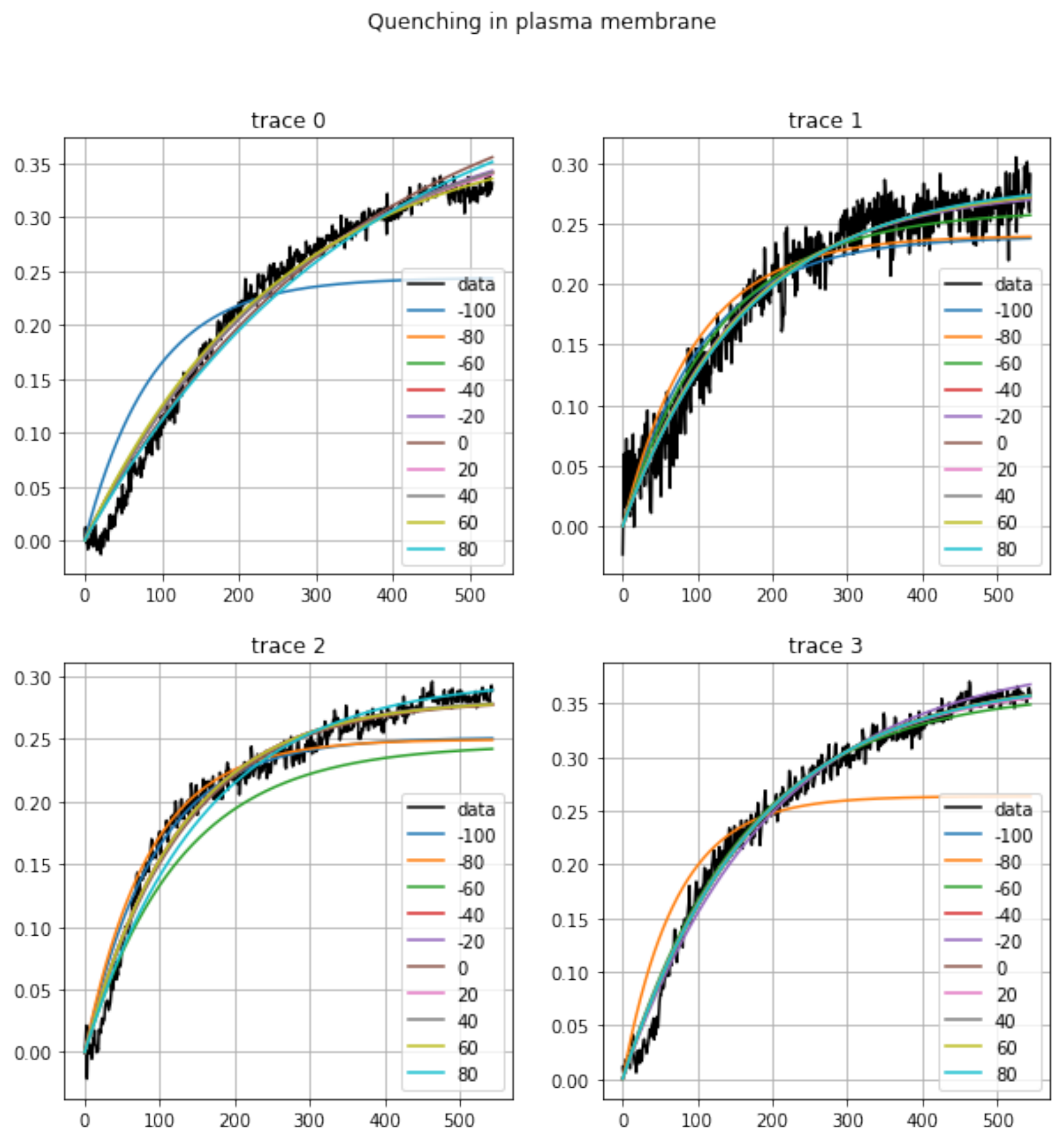

Runing the model¶

In [48]:

```
"""Since there is no effect of voltage, we averages
rate constants
obtainded from indivual fits to run the model
"""

a_fit = np.mean(a)
b_fit = np.mean(b)
# same model used before
```

```
def ff(z, t, a,b):  # Model , a and b are rate
constant to be fitted
    c1,m1,m2 = z   # Probability to be in each
compartment, can be transformed in concentration
    A0 = 10643.1
    slp_a = -.215
    V = -30  # membrane potential
    v2 = A0*np.exp(-slp_a*V/58) # .002 # voltage
dependent rate constant
    B0 = 8624.9
    slp_b = .278 # 1.278
    v1 = B0*np.exp((1-slp_b)*V/58) # .002 # voltage
dependent rate constant

    dydt = [-a*c1 + b*m1,               # C1
        a*c1 + v2*m2 - b*m1 - v1*m1, # M1
        v1*m1 + a*(1- c1 -m1 -m2) - v2*m2 -b*m2]  #
M2
        #b*m2 -a*(1- c1 -m1 -m2)]     # Cyto
    return dydt

y0=[1,0,0] #initial values
t_trazos= np.arange(0, 2000, 1)   # time array
# Solve ODE
y_fit = integrate.odeint(ff, y0, t=t_trazos,
args=(a_fit,b_fit))
# each column of y_fit is one compartment: c1, m1,
m2, 1-c1-m1-m2 (c2,cytosol)
y_fit_comp = 1- y_fit[:,0] -y_fit[:,1] -y_fit[:,2]
# this is c2
plt.figure(figsize=(10,5))

#plot all probabilities
plt.subplot(1,2,1)
#plt.plot(t_trazos, y_fit[:,0], "k", label ="c1")
plt.plot(t_trazos, y_fit[:,1], "b", label ="m1")  #
plot m1
plt.plot(t_trazos, y_fit[:,2], "g", label ="m2")  #
```

```
plot c2 (cytosol)
plt.plot(t_trazos, y_fit[:,2] + y_fit[:,1], "--g",
label ="sum")  # plot c2 (cytosol)
plt.plot(t_trazos, y_fit_comp, "r", label ="1-c1-
m1-m2")  # plot m2
plt.axvline(900, ls="--", color="k")
V_m =12 # m^3
#magia_m1 = ((moles_m1+ moles_m2/V_m) * (y_fit[:,1]
+ y_fit[:,2])  # M1
#magia_cyt = (moles_cyt/v_cyt)*y_fit_comp)

plt.legend(loc=4)
plt.xlabel("time")
plt.ylabel("Probability")
plt.grid()

#plot all normalized probabilities
plt.subplot(1,2,2)
#plt.plot(t_trazos, y_1[:,0]/np.amax(y_1[:,0]) ,
"k", label ="c1")
plt.plot(t_trazos, y_fit[:,1]/np.amax(y_fit[:,1]),
"b", label ="m1")
plt.plot(t_trazos, y_fit[:,2]/np.amax(y_fit[:,2]),
"g", label ="m2")
plt.plot(t_trazos, y_fit_comp/np.amax(y_fit_comp),
"r", label ="1-c1-m1-m2")

plt.legend(loc=4)
plt.xlabel("time")
plt.ylabel("probability/norm at max")
plt.suptitle("Four compartment model, two rates")
plt.grid()
```

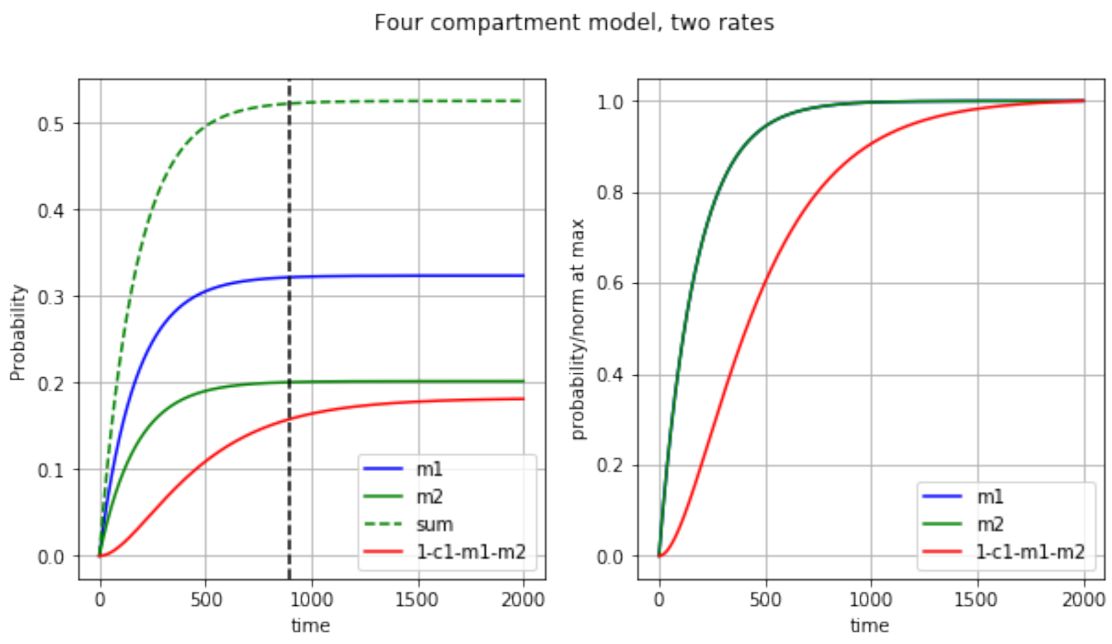

DPA concentration in plasma membrane¶

By using density of DPA in plasma membrane in similar conditions (10**-4 molecules / Å^2), the volume of a spherical cell of 20 micrometers diameter and 10 nm membrane thickness, we calculate maximum DPA concentration in membrane.

In [8]:

```
# Number of molecules in membrane
density = 10**-4 # molec / Angs^2
ra = 20 # um
s_a  = 4*math.pi*((ra*10000 )**2) # surface in
Angstrong^2
nu_molec = density*s_a
print ("# molec per cell %.0f" %nu_molec)
# vol cell
vol_cell_um = 4*math.pi*(ra**3)/3 # in um^3
print ("cell volume is %.1f" %vol_cell_um  +"
um^3")
# vol membrane
ra_m = .01 + ra  # radius 10 nm bigger than ra
vol_cell_um_m = 4*math.pi*(ra_m**3)/3 # in um^3
vol_mem = vol_cell_um_m - vol_cell_um
print ("mem  volume is %.1f" %vol_mem  +" um^3")
```

```
vol_cyto = .3*vol_cell_um
print ("cyto volume is %.1f" %vol_cyto  +" um^3")
print ("--------")
# DPA conc in membrane
avo = 6.023*10**23
dpa_pm = (nu_molec/avo)/(vol_cell_um*10**-15)  #
*10**15 convertion um3 to L
print("[DPA]_pm " + str(dpa_pm) +"M" ) # %.1f
```

```
# molec per cell 50265482
cell volume is 33510.3 um^3
mem  volume is 50.3 um^3
cyto volume is 10053.1 um^3
-------
[DPA]_pm 2.49045326249e-06M
```

DPA concentration in compartments¶

By using three simple rule with the maximum DPA concentration in plasma membrane, probabilities were transformed probabilities into number of molecules. Concentration was obtained by dividing by Avogadros number and subsecuently by membrane volume.

In [51]:

```
# calculate number of molecules
mem_ss = y_fit[-1,1] + y_fit[-1,2]  # M1 + M2 at
steady state
# number of molecules calculated by rule of three
using known density
#  mem_ss (0.55)   -> num_molec in membrane
(12566371)
#    array          ->    x
# x = array * number_of_molecules / mem_ss
m1_num_molec = y_fit[:,1]*nu_molec/mem_ss
m2_num_molec = y_fit[:,2]*nu_molec/mem_ss
cyto_num_molec = y_fit_comp*nu_molec/mem_ss

# calculate concentration
#  [] = (num_molec/avogadro)/vol (in liters)
```

```
m1_conc = (m1_num_molec/avo)/(vol_mem*10**-15)
m2_conc = (m2_num_molec/avo)/(vol_mem*10**-15)
mem_conc = ((m1_num_molec +m2_num_molec)/avo)/
(vol_mem*10**-15)
vol_cyto = .4*(vol_cell_um*10**-15)  # cytoplasm is
one third of cell volume
cyt_conc = (cyto_num_molec/avo)/(vol_cyto)

# Plot probability
fig = plt.figure(figsize=(12,5))
plt.subplot(1,3,1)
#plt.plot(t_trazos, y_fit[:,0], "k", label ="c1")
plt.plot(t_trazos, y_fit[:,1], "b", label ="m1")  #
plot m1
plt.plot(t_trazos, y_fit[:,2], "g", label ="m2")  #
plot c2 (cytosol)
plt.plot(t_trazos, y_fit[:,2] + y_fit[:,1], "--g",
label ="m1 + m2")  # plot c2 (cytosol)
plt.plot(t_trazos, y_fit_comp, "r", label ="cyto")
# plot m2
plt.axvline(900, ls="--", color="k")
plt.ylabel("Probability")
plt.legend(loc=1)
plt.xlabel("time")
plt.grid()

# Plot number of molecules
plt.subplot(1,3,2)
#plt.plot(t_trazos, y_fit[:,0], "k", label ="c1")
plt.plot(t_trazos, m1_num_molec, "b", label ="m1")
# plot m1
plt.plot(t_trazos, m2_num_molec, "g", label ="m2")
# plot c2 (cytosol)
plt.plot(t_trazos, m1_num_molec + m2_num_molec, "--
g", label ="m1 + m2")  # plot c2 (cytosol)
plt.plot(t_trazos, cyto_num_molec, "r", label
="cyto")  # plot m2
plt.axvline(900, ls="--", color="k")
```

```
plt.ylabel("Number of molecules")
plt.legend(loc=1)
plt.xlabel("time")
plt.grid()

# Plot concentration
ax1 = plt.subplot(1,3,3)
#plt.plot(t_trazos, y_fit[:,0], "k", label ="c1")
ax1.plot(t_trazos, m1_conc, "b", label ="m1")  #
plot m1
ax1.plot(t_trazos, m2_conc, "g", label ="m2")  #
plot c2 (cytosol)
ax1.plot(t_trazos, mem_conc, "--g", label ="m1 +
m2")  # plot c2 (cytosol)
ax1.plot(t_trazos, cyt_conc, "r", label ="cyto")  #
plot m2
ax1.set_xlabel("time")
ax1.set_ylabel("DPA Concentration (M)")
ax1.ticklabel_format(axis="y", style="sci",
scilimits=(0,0))
ax1.axvline(900, ls="--", color="k")
ax1.set_ylabel("DPA Concentration (M)")
ax1.legend(loc=1)
ax1.set_xlabel("time")
ax1.grid()
#ax1.axvline(520, ls="--", color="r")
[x0,y0], [x1, y1] =
fig.transFigure.inverted().transform(
            ax1.transAxes.transform([[0.8, 0.05],
[1.3, 0.35]]))
ax2 = fig.add_axes([x0, y0, x1-x0, y1-y0 ])
ax2.plot(t_trazos, cyt_conc, "r", label ="cyto")  #
plot m2
ax2.ticklabel_format(axis="y", style="sci",
scilimits=(0,0))
ax2.axvline(900, ls="--", color="k")
ax2.grid()
#for i in range(3):
```

```python
#    plt.subplot(1,3,i+1)
#    plt.legend(loc=1)
#    plt.xlabel("time")
#    plt.grid()
print("concentration at 900 s")
print("conc in PM "+ str(mem_conc[899])+ " M")
print("conc in cyto "+ str(cyt_conc[899])+ " M")
#print("conc in lyso lumen "+ mem_conc[-1])
plt.tight_layout()

plt.savefig("probs_and_concs.png", dpi=300)
```

```
concentration at 900 s
conc in PM 0.0016501459493953171 M
conc in cyto 1.8733943055539614e-06 M
```

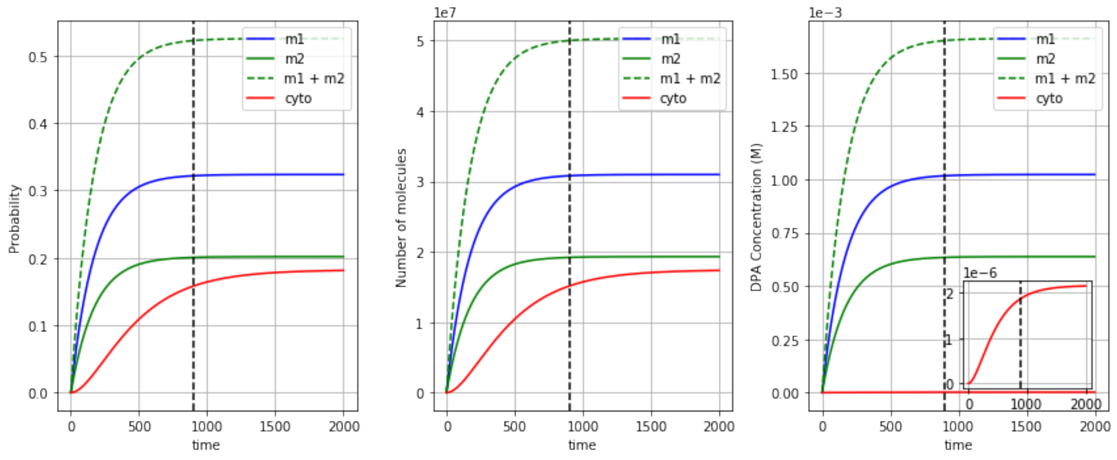

In [50]:

```python
print(np.shape(t_trazos))
```

```
(2000,)
```

Plot extracellular, membrane (m1 + m2) and cytosol

In [52]:

```python
# Plot probability
fig = plt.figure(figsize=(12,5))
plt.subplot(1,3,1)
plt.plot(t_trazos, y_fit[:,0], "k", label ="ext")
#plt.plot(t_trazos, y_fit[:,1], "b", label ="m1")
```

```
# plot m1
#plt.plot(t_trazos, y_fit[:,2], "g", label ="m2")
# plot c2 (cytosol)
plt.plot(t_trazos, y_fit[:,2] + y_fit[:,1], "--g",
label ="membrane")  # plot c2 (cytosol)
plt.plot(t_trazos, y_fit_comp, "r", label ="cyto")
# plot m2
plt.axvline(900, ls="--", color="k")
plt.ylabel("Probability")
plt.legend(loc=1)
plt.xlabel("time")
#plt.grid()

# Plot number of molecules
ext_num_molec = y_fit[:,0]*nu_molec/mem_ss
plt.subplot(1,3,2)
plt.plot(t_trazos, ext_num_molec, "k", label
="ext")
#plt.plot(t_trazos, m1_num_molec, "b", label ="m1")
# plot m1
#plt.plot(t_trazos, m2_num_molec, "g", label ="m2")
# plot c2 (cytosol)
plt.plot(t_trazos, m1_num_molec + m2_num_molec, "--
g", label ="membrane")  # plot c2 (cytosol)
plt.plot(t_trazos, cyto_num_molec, "r", label
="cyto")  # plot m2
plt.axvline(900, ls="--", color="k")
plt.ylabel("Number of molecules")
plt.legend(loc=1)
plt.xlabel("time")
#plt.grid()

# Plot concentration
ax1 = plt.subplot(1,3,3)
#plt.plot(t_trazos, y_fit[:,0], "k", label ="c1")
#plt.plot(t_trazos, m1_conc, "b", label ="m1")  #
plot m1
#plt.plot(t_trazos, m2_conc, "g", label ="m2")  #
```

```
plot c2 (cytosol)
ax1.plot(t_trazos, mem_conc, "--g", label
="membrane")  # plot c2 (cytosol)
#ax1.plot(t_trazos, cyt_conc, "r", label ="cyto")
# plot m2
ax1.legend(loc=4)
ax1.set_xlabel("time")
ax1.set_ylabel("DPA Concentration (M)")
ax1.ticklabel_format(axis="y", style="sci",
scilimits=(0,0))
ax1.axvline(900, ls="--", color="k")
[x0,y0], [x1, y1] =
fig.transFigure.inverted().transform(
            ax1.transAxes.transform([[0.8, 0.05],
[1.3, 0.35]]))
ax1.legend(loc=1)
ax1.set_xlabel("time")
#ax1.grid()
ax2 = fig.add_axes([x0, y0, x1-x0, y1-y0 ])
ax2.plot(t_trazos, cyt_conc, "r", label ="cyto")  #
plot m2
ax2.ticklabel_format(axis="y", style="sci",
scilimits=(0,0))
ax2.axvline(900, ls="--", color="k")
#ax2.grid()

plt.tight_layout()
```

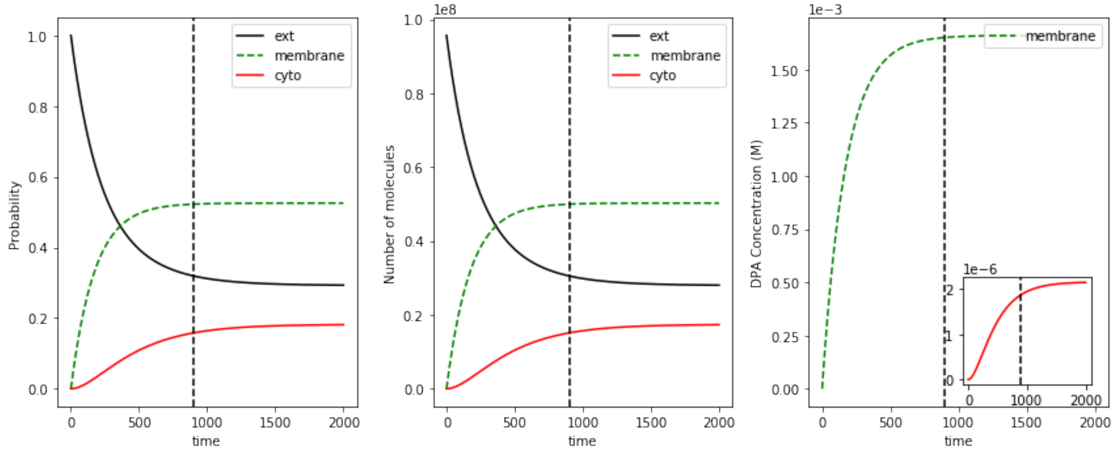

Effect of cytoplasm proportion on concentration.¶

Calculate concentration on different proportions of cytoplasm volume/cell volume

In [54]:

```
# Effect of cytoplasm proprtion on concentration
props = [.2, .3, .4, .5 ]
l_s = [":", "-", "--", "-."]
#plt.plot(t_trazos, mem_conc, "--g", label ="m1 + m2")  # plot c2 (cytosol)
#plt.figure(figsize=(6,6))
fig,ax = plt.subplots(figsize=(6,6))
print("concentrations in cytosol at 900 s")
for i in range(len(props)):
    vol_cyto = props[i]*(vol_cell_um*10**-15)  # cytoplasm is one third of cell volume
    cyt_conc = (cyto_num_molec/avo)/(vol_cyto)
    ax.plot(t_trazos, cyt_conc, "r", ls=l_s[i], label =str(props[i]))  #
    print("proportion " +str(props[i]) +" conc in cyto is "+ str(cyt_conc[899]))
#ax.set_ylim(0,1e-5)
ax.set_xlim(0,2000)
ax.legend(loc=4)
ax.set_xlabel("Time (s)", fontsize=16)
ax.set_ylabel("DPA Concentration (M)", fontsize=16)
ax.ticklabel_format(axis="y", style="sci", scilimits=(0,0))
ax.axvline(900, ls="--", color="k")
# axis
ax.axvline( linewidth =4, color="k")
ax.axhline( linewidth =3, color="k")
ax.spines["top"].set_visible(False)
ax.spines["right"].set_visible(False)
ax.tick_params(axis="both", width=2, length=4, labelsize=14)

plt.savefig("cyt_concentration.png", dpi=300)
```

```
plt.savefig("cyt_concentration.pdf", dpi=300)
```

```
concentrations in cytosol at 900 s
proportion 0.2 conc in cyto is
3.7467886111079227e-06
proportion 0.3 conc in cyto is
2.4978590740719485e-06
proportion 0.4 conc in cyto is
1.8733943055539614e-06
proportion 0.5 conc in cyto is
1.4987154444431692e-06
```

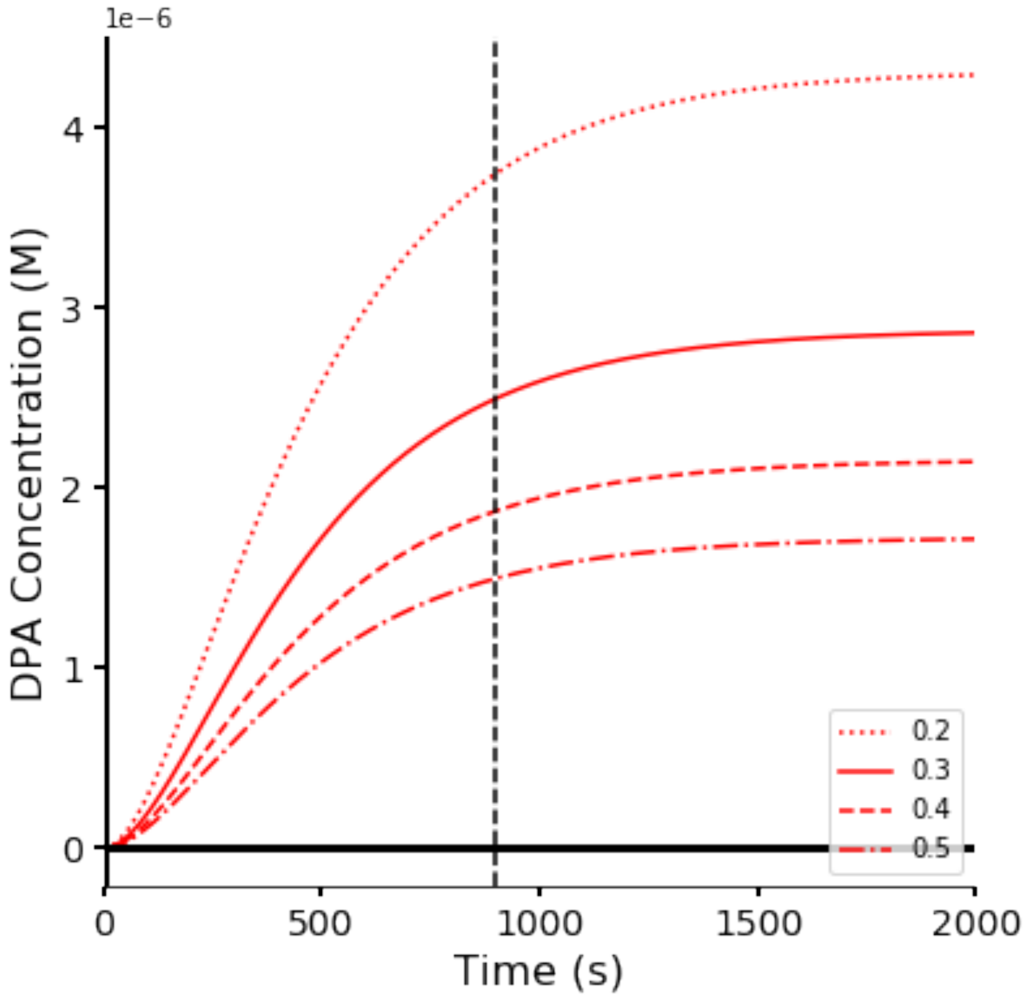

Concentrations in Lysosome¶

Model with lysosomal membranes and lumen

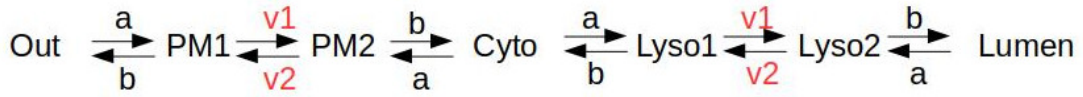

In [63]:

```
a_fit = np.mean(a)
b_fit = np.mean(b)
def ff2(z, t, a,b):  # Model , a and b are rate
constant to be fitted
    c1,m1,m2,l1,l2,u = z    # Probability to be in
each compartment, can be transformed in
concentration
    A0 = 10643.1
    slp_a = -.215
    V = -30  # membrane potential
    v2 = A0*np.exp(-slp_a*V/58) # .002 # voltage
dependent rate constant
    B0 = 8624.9
    slp_b = .278 # 1.278
    v1 = B0*np.exp((1-slp_b)*V/58) # .002 # voltage
dependent rate constant

    #dydt = [-a*c1 + b*m1,             # C1
    #    a*c1 + v2*m2 - b*m1 - v1*m1, # M1
    #    v1*m1 + a*(1- c1 -m1 -m2) - v2*m2 -b*m2]
# M2
    #   #b*m2 -a*(1- c1 -m1 -m2)]     # Cyto

    dydt = [-a*c1 + b*m1, # C1
        a*c1 + v2*m2 - b*m1 - v1*m1, # M1
        v1*m1 + a*(1- c1 -m1 -m2 -l1 -l2 -u) -
v2*m2 -b*m2,  # M2
        #b*m2 + b*l1 -2*a*(1- c1 -m1 -m2-l1 -l2
-u),    # C3  cyto
        a*(1- c1 -m1 -m2 -l1 -l2 -u) +v2*l2 -b*l1 -
v1*l1,    # L1
        v1*l1 -v2*l2 + a*u - b*l2,  # L2
```

```
        b*l2 - a*u]    #lyso

    return dydt

y0=[1,0,0,0,0,0] #initial values
t_trazos= np.arange(0, 2000, 1)   # time array
# Solve ODE
y_fit = integrate.odeint(ff2, y0, t=t_trazos,
args=(a_fit,b_fit))
# each column of y_fit is one compartment: c1, m1,
m2, 1-c1-m1-m2 (c2,cytosol)
y_fit_comp = 1- y_fit[:,0] -y_fit[:,1] -y_fit[:,2]
-y_fit[:,3] -y_fit[:,4] -y_fit[:,5]# this is cyto

plt.figure(figsize=(10,5))
plt.plot(t_trazos, y_fit[:,1], "-b", label ="m1")
# plot m1
plt.plot(t_trazos, y_fit[:,2], "--b", label ="m2")
# plot c2 (cytosol)
#plt.plot(t_trazos, y_fit[:,2] + y_fit[:,1], "--g",
label ="sum M")  # plot c2 (cytosol)
plt.plot(t_trazos, y_fit_comp, "r", label ="cyto")
# plot cyto
plt.plot(t_trazos, y_fit[:,3], "-g", label ="l1")
# plot l1
plt.plot(t_trazos, y_fit[:,4], "--g", label ="l2")
# plot l2
plt.plot(t_trazos, y_fit[:,5], "gray", label
="lyso")  # plot lyso
plt.axvline(900, ls="--", color="k")
plt.legend()
```

Out[63]:

```
<matplotlib.legend.Legend at 0x7fced62ba410>
```

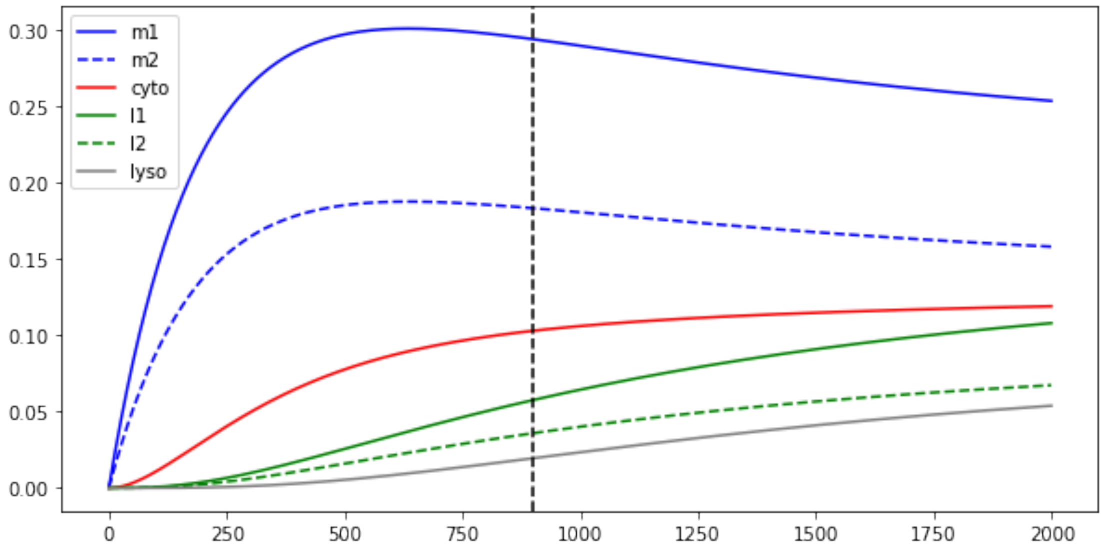

Calculating concentration in each compartment

In [64]:

```
# calculate number of molecules
mem_ss = y_fit[-1,1] + y_fit[-1,2]  # M1 + M2 at
steady state
# number of molecules calculated by rule of three
using known density
#  mem_ss (0.55)   -> num_molec in membrane
(12566371)
#    array          ->    x
# x = array * number_of_molecules / mem_ss
m1_num_molec_2 = y_fit[:,1]*nu_molec/mem_ss
m2_num_molec_2 = y_fit[:,2]*nu_molec/mem_ss
cyto_num_molec_2 = y_fit_comp*nu_molec/mem_ss
l1_num_molec_2 = y_fit[:,3]*nu_molec/mem_ss
l2_num_molec_2 = y_fit[:,4]*nu_molec/mem_ss
ly_num_molec_2 = y_fit[:,5]*nu_molec/mem_ss



# calculate concentration
#  [] = (num_molec/avogadro)/vol (in liters)
m1_conc_2 = (m1_num_molec_2/avo)/(vol_mem*10**-15)
m2_conc_2 = (m2_num_molec_2/avo)/(vol_mem*10**-15)
```

```
mem_conc_2 = ((m1_num_molec_2 +m2_num_molec_2)/
avo)/(vol_mem*10**-15)
vol_cyto = .4*(vol_cell_um*10**-15)  # cytoplasm is
one third of cell volume
cyt_conc_2 = (cyto_num_molec_2/avo)/(vol_cyto)

# vol lysosome
ra_l = .5  # radius lyso 0.5 micro meter
vol_lyso_um = 4*math.pi*(ra_l**3)/3 # in um^3
ra_l_m = .01 + ra_l  # radius 10 nm bigger than ra
vol_lyso_um_m = 4*math.pi*(ra_l_m**3)/3 # in um^3
vol_mem_lyso = vol_lyso_um_m - vol_lyso_um
#print ("mem  volume is %.1f" %vol_mem  +" um^3")

vol_cyto = .3*vol_cell_um

l1_conc = (l1_num_molec_2/avo)/(vol_mem*10**-15)
l2_conc = (l2_num_molec_2/avo)/(vol_mem*10**-15)
ly_mem_conc = l1_conc + l2_conc   #((l1_num_molec_2
+l2_num_molec_2)/avo)/(vol_mem*10**-15)
ly_conc = (ly_num_molec_2/avo)/(vol_mem*10**-15)

# Plot probability
fig = plt.figure(figsize=(12,5))
plt.subplot(1,3,1)
#plt.plot(t_trazos, y_fit[:,0], "k", label ="c1")
plt.plot(t_trazos, y_fit[:,1], "b", label ="m1")  #
plot m1
plt.plot(t_trazos, y_fit[:,2], "--b", label ="m2")
# plot c2 (cytosol)
plt.plot(t_trazos, y_fit_comp, "r", label ="cyto")
# plot cyto
plt.plot(t_trazos, y_fit[:,3], "-g", label ="l1")
# plot l1
plt.plot(t_trazos, y_fit[:,4], "--g", label ="l2")
# plot l2
plt.plot(t_trazos, y_fit[:,5], "gray", label
="lyso")  # plot lyso
```

```
#plt.plot(t_trazos, y_fit[:,2] + y_fit[:,1], "--g",
label ="m1 + m2")  # plot c2 (cytosol)
#plt.plot(t_trazos, y_fit_comp, "r", label ="cyto")
# plot m2
plt.axvline(900, ls="--", color="k")
plt.ylabel("Probability")
plt.legend(loc=1)
plt.xlabel("time (s)")
plt.grid()

# Plot number of molecules
plt.subplot(1,3,2)
#plt.plot(t_trazos, y_fit[:,0], "k", label ="c1")
plt.plot(t_trazos, m1_num_molec_2, "b", label
="m1")  # plot m1
plt.plot(t_trazos, m2_num_molec_2, "--b", label
="m2")  # plot c2 (cytosol)
#plt.plot(t_trazos, m1_num_molec + m2_num_molec,
"--g", label ="m1 + m2")  # plot c2 (cytosol)
plt.plot(t_trazos, cyto_num_molec_2, "r", label
="cyto")  # plot m2
plt.plot(t_trazos, l1_num_molec_2, "g", label
="l1")  # plot c2 (cytosol)
plt.plot(t_trazos, l2_num_molec_2, "--g", label
="l2")  # plot c2 (cytosol)
plt.plot(t_trazos, ly_num_molec_2, "gray", label
="ly")  # plot c2 (cytosol)

plt.axvline(900, ls="--", color="k")
plt.ylabel("Number of molecules")
plt.legend(loc=1)
plt.xlabel("time (s)")
plt.grid()

# Plot concentration
ax1 = plt.subplot(1,3,3)
#plt.plot(t_trazos, y_fit[:,0], "k", label ="c1")
ax1.plot(t_trazos, m1_conc_2, "b", label ="m1")  #
```

```
plot m1
ax1.plot(t_trazos, m2_conc_2, "--b", label ="m2")
# plot c2 (cytosol)
ax1.plot(t_trazos, mem_conc_2, ":b", label
="m1+m2")  # plot c2 (cytosol)
ax1.plot(t_trazos, cyt_conc_2, "r", label ="cyto")
# plot m2
ax1.plot(t_trazos, l1_conc, "g", label ="l1")  #
plot c2 (cytosol)
ax1.plot(t_trazos, l2_conc, "--g", label ="l2")  #
plot c2 (cytosol)
ax1.plot(t_trazos, ly_mem_conc, ":g", label
="l1+l2")  # plot c2 (cytosol)
ax1.plot(t_trazos, ly_conc, color="gray", label
="ly")  # plot c2 (cytosol)

ax1.set_xlabel("time")
ax1.set_ylabel("DPA Concentration (M)")
ax1.ticklabel_format(axis="y", style="sci",
scilimits=(0,0))
ax1.axvline(900, ls="--", color="k")
ax1.set_ylabel("DPA Concentration (M)")
ax1.legend(loc=1)
ax1.set_xlabel("time (s)")
ax1.grid()
#ax1.axvline(520, ls="--", color="r")
[x0,y0], [x1, y1] =
fig.transFigure.inverted().transform(
            ax1.transAxes.transform([[0.35, 0.7],
[.85, 1.0]]))
            # [0.8, 0.05], [1.3, 0.35]

ax2 = fig.add_axes([x0, y0, x1-x0, y1-y0 ])
ax2.plot(t_trazos, cyt_conc_2, "r", label ="cyto")
# plot m2
ax2.ticklabel_format(axis="y", style="sci",
scilimits=(0,0))
ax2.axvline(900, ls="--", color="k")
```

```
#ax2.grid()
ax2.patch.set_alpha(.5)
ax2.legend(loc=4)

#for i in range(3):
#    plt.subplot(1,3,i+1)
#    plt.legend(loc=1)
#    plt.xlabel("time")
#    plt.grid()

print("conc in PM \t\t"+ str(mem_conc_2[899])+ " M")
print("conc in cyto \t\t"+ str(cyt_conc_2[899])+ " M")
print("conc in lyso mem \t"+ str(ly_mem_conc[899])+ " M")
print("conc in lumen lyso \t"+ str(ly_conc[899])+ " M")
#print("conc in cyto "+ str(cyt_conc[-1])+ " M")

plt.tight_layout()
plt.savefig("probs_and_concs_pm_ly.png", dpi=300)
```

```
conc in PM 		0.0019242381541639706 M
conc in cyto 		1.5574334854629833e-06 M
conc in lyso mem 	0.00037672837673175343 M
conc in lumen lyso 	7.842249488728324e-05 M
```

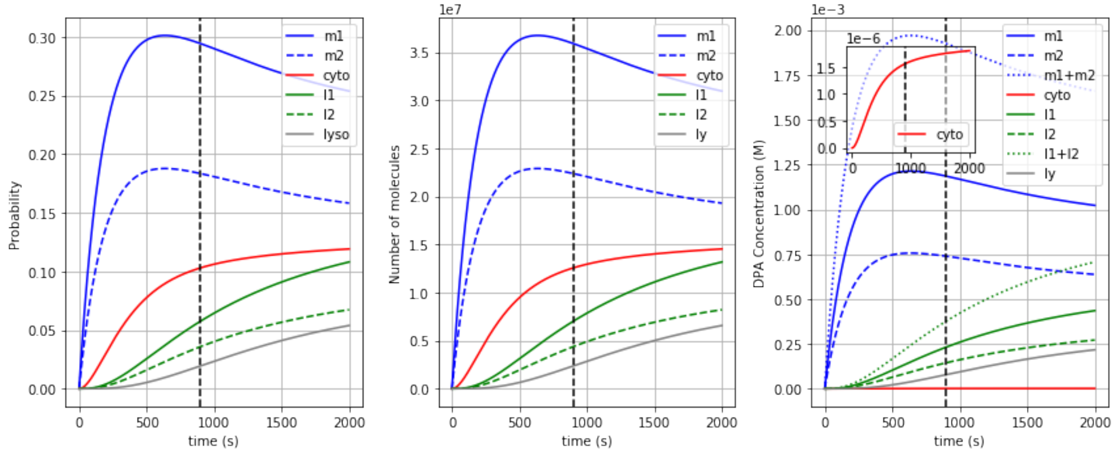

Supplement: Supplementary file 6 — Supplementary Data 1 [file 42003_2021_1916_MOESM6_ESM.pdf]
