# Supplementary material for: Imaging the electrical activity of organelles in living cells
Source: Commun Biol. 2021 Mar 23;4:389. doi: 10.1038/s42003-021-01916-6 (PMC7988155; doi:10.1038/s42003-021-01916-6)
Supplement: Supplementary file 8 — Reporting Summary [file 42003_2021_1916_MOESM8_ESM.pdf]

nature research

Corresponding author(s): Sebastian Brauchi

Last updated by author(s): Dec 11, 2020

Reporting Summary

Nature Research wishes to improve the reproducibility of the work that we publish. This form provides structure for consistency and transparency in reporting. For further information on Nature Research policies, see our Editorial Policies and the Editorial Policy Checklist.

Statistics

For all statistical analyses, confirm that the following items are present in the figure legend, table legend, main text, or Methods section.

n/a	Confirmed	
☐	☒	The exact sample size (*n*) for each experimental group/condition, given as a discrete number and unit of measurement
☐	☒	A statement on whether measurements were taken from distinct samples or whether the same sample was measured repeatedly
☐	☒	The statistical test(s) used AND whether they are one- or two-sided<br>*Only common tests should be described solely by name; describe more complex techniques in the Methods section.*
☐	☒	A description of all covariates tested
☐	☒	A description of any assumptions or corrections, such as tests of normality and adjustment for multiple comparisons
☐	☒	A full description of the statistical parameters including central tendency (e.g. means) or other basic estimates (e.g. regression coefficient) AND variation (e.g. standard deviation) or associated estimates of uncertainty (e.g. confidence intervals)
☐	☒	For null hypothesis testing, the test statistic (e.g. *F*, *t*, *r*) with confidence intervals, effect sizes, degrees of freedom and *P* value noted<br>*Give P values as exact values whenever suitable.*
☒	☐	For Bayesian analysis, information on the choice of priors and Markov chain Monte Carlo settings
☒	☐	For hierarchical and complex designs, identification of the appropriate level for tests and full reporting of outcomes
☒	☐	Estimates of effect sizes (e.g. Cohen's *d*, Pearson's *r*), indicating how they were calculated

Our web collection on statistics for biologists contains articles on many of the points above.

Software and code

Policy information about availability of computer code

Data collection	Data was collected in three different platforms. Voltage and pH imaging: Image acquisition was controlled by micro-manager 1.4 (Open Imaging, San Francisco, CA). Space-correlated imaging: Image acquisition was controlled by SlideBook6 (Intelligent Imaging Innovations). Optogenetics: Imaging and photo-stimulation was controlled by Zen 3.1 software (Zeiss)
Data analysis	All numerical data was treated, and the statistical analysis was computed using Microcal OriginPro ver9 (OriginLab corporation). Figures were prepared using Microcal OriginPro, ImageJ, Biorender, and Inkscape. The mathematical model was written in Python language and implemented in Jupiter notebook. All the data, analysis, materials, and code contained in this study is available from the corresponding author upon request.

For manuscripts utilizing custom algorithms or software that are central to the research but not yet described in published literature, software must be made available to editors and reviewers. We strongly encourage code deposition in a community repository (e.g. GitHub). See the Nature Research guidelines for submitting code & software for further information.

Data

Policy information about availability of data

All manuscripts must include a data availability statement. This statement should provide the following information, where applicable:

- Accession codes, unique identifiers, or web links for publicly available datasets
- A list of figures that have associated raw data
- A description of any restrictions on data availability

All the data, analysis, materials, and code contained in this study is available from the corresponding author upon request.

Field-specific reporting

Please select the one below that is the best fit for your research. If you are not sure, read the appropriate sections before making your selection.

☒ Life sciences ☐ Behavioural & social sciences ☐ Ecological, evolutionary & environmental sciences

For a reference copy of the document with all sections, see nature.com/documents/nr-reporting-summary-flat.pdf

Life sciences study design

All studies must disclose on these points even when the disclosure is negative.

Sample size	Sample size of each measurement was determined to an adequate representation of the population. No statistical estimation of sample size was performed
Data exclusions	In exceptional cases some replicates (ROIs) were excluded from the final analysis after inspection of the whole dataset by GraphPad Outlier Calculator (https://www.graphpad.com/quickcalcs/Grubbs1.cfm).
Replication	The approach was applied to multiple independent datasets, as presented in the manuscript. Multiple batches of cell-line served as replicates and repeated at different time-points (showing consistency in all dataset).
Randomization	Culture dishes containing various cell types were randomized during imaging experiments. Individual cells were randomly selected during experiments.
Blinding	We did not use blind experiments in this present work.

Reporting for specific materials, systems and methods

We require information from authors about some types of materials, experimental systems and methods used in many studies. Here, indicate whether each material, system or method listed is relevant to your study. If you are not sure if a list item applies to your research, read the appropriate section before selecting a response.

Materials & experimental systems

n/a	Involved in the study	
☒	☐	Antibodies
☐	☒	Eukaryotic cell lines
☒	☐	Palaeontology and archaeology
☒	☐	Animals and other organisms
☒	☐	Human research participants
☒	☐	Clinical data
☒	☐	Dual use research of concern

Methods

n/a	Involved in the study	
☒	☐	ChIP-seq
☒	☐	Flow cytometry
☒	☐	MRI-based neuroimaging

Eukaryotic cell lines

Policy information about cell lines

Cell line source(s)	In this study we used: HEK293; MCF7; U2OS; PC-12 cells. HEK293 was directly obtained from ATCC. PC12 cells belongs to our own laboratory batch originally obtained from Boston Children's Hospital. MCF7 and U2OS were obtained form Janelia RC cell culture facility.
Authentication	Non of the cell line were authenticated.
Mycoplasma contamination	We routinely check our cell lines for Mycoplasma contamination by PCR and all cell lines used in this study turned out to be negative for Mycoplasma contamination.
Commonly misidentified lines (See ICLAC register)	We used only common and easily identifiable cell lines.
